# Green nanotechnology for the enhancement of antibacterial properties in lining leather: MgO-chitosan nanocomposite coating

**DOI:** 10.1016/j.heliyon.2024.e39170

**Published:** 2024-10-12

**Authors:** Sobur Ahmed, Sajib Sarker Imon, Md Jawad Hasan, Md Samaul Alam

**Affiliations:** Department of Leather Engineering, Institute of Leather Engineering and Technology, University of Dhaka, 44-50, Hazaribagh, Dhaka-1209, Bangladesh

**Keywords:** *Aloe vera* extract, Antimicrobial finish, Bio-based nanocomposite, Cytotoxicity, Lining leather

## Abstract

Antimicrobial nanomaterials have received a lot of interest in recent years due to their potential to fight against microbial degradation, a common problem in leather products. In this study, a nanocomposite was synthesized with MgO nanoparticles prepared by Aloe vera leaf extract and chitosan (CS), as an innovative solution to this problem. Three nanocomposite samples (C1, C2, and C3) were formulated with varying ratios of MgO and chitosan and evaluated for antimicrobial efficacy against *Escherichia coli* and Bacillus subtilis. Leather treated with MgO/Chitosan nanocomposite (MgO/Chitosan-1:1) exhibited substantial inhibition zones of 13 mm and 12 mm against *E. coli* and *B. subtilis*, respectively. Characterization of MgO nanoparticles, chitosan, and MgO/CS nanocomposite was performed through FTIR, XRD, SEM, TGA, and cytotoxicity tests. The average particle size and crystallite size of MgO nanoparticles were found as 136 nm and 10.3 nm, respectively and a weight loss of 67 % for MgO/CS nanocomposite in thermogravimetric analysis. FTIR confirmed the successful incorporation of MgO nanoparticles into the chitosan matrix, evidenced by the presence of characteristic functional groups. Application of nanocomposite onto lining leather via spraying resulted in finished leather with improved color rub fastness, perspiration fastness, and thermal stability compared to untreated leather. In comparison to dry color rub fastness, wet color rub fastness was notably improved by the MgO/CS nanocomposite, with gray scale ratings ranging from 4/5 to 5. Perspiration fastness was marginally enhanced by the MgO/CS coating, with gray scale ratings ranging from 4/5 to 5 for both grain and flesh samples. Specifically, the coated leather exhibited a water vapor permeability (WVP) of 9.94 mg cm^−2^.hr^−1^ that was lower than both uncoated (12.37 mg cm^−2^.hr^−1^) and PVA-coated (11.22 mg cm^−2^.hr^−1^) leather. This study presents a promising solution to the challenge of microbial degradation in leather products and highlights the potential of natural sources for synthesizing functional nanocomposites with diverse applications in materials science and biotechnology.

## 1 Introduction

Footwear and clothing have historically been among the most fundamental human needs. Animal skins were used in the past by humans to satisfy their needs. Leather, a natural material produced from animal hides or skins, is extensively used for manufacturing a wide range of products, including shoes, purses, wallets, apparel, and other accessories. Leather is comfortable to wear because of its outstanding moisture-absorbing capacity, breathability, and flexibility [[1], [2], [3]]. Due to the presence of functional groups like –NH_2_, –COO–, and –OH [4], leather is inherently hydrophilic and provides an ideal environment for the growth of bacteria, mildew, and moulds that cause skin problems, foul odors, and discomfort to the user [5,6]. The collagen matrix of the leather structure offers the right amount of oxygen, moisture, and temperature for the growth of microorganisms. All living things are fatally affected by microbial infections. The smell coming from our shoes, socks, and/or feet is caused by *Staphylococcus epidermidis* and *Brevibacterium linens* breaking down the amino acids in our perspiration and skin. *B. linens* can convert the amino acid methionine found in perspiration into the gas methanethiol. By converting perspiration into isovaleric acid (3-methyl butanoic acid) from the amino acid leucine, *S. epidermidis* produces body odor [7]. Proteins found in perspiration may function as a nutrient supply for the growth of fungi and bacteria on leather products because of their high moisture absorption capacity, especially in shoes where the lining surface and the foot skin remain in close contact [[8], [9], [10], [11], [12]]. The bacteria *Staphylococcus epidermidis* converts leucine in perspiration to isovaleric acid, which produces malodor in footwear and feet [8].

Leather provides an ideal environment for the development and spread of microbes, which is necessary to be treated with substances for protection against the growth of bacteria. Metal nanoparticles can improve leather's characteristics like flame retardancy, abrasion resistance, self-cleaning, and antimicrobial resistance by improving its natural qualities [13]. The hot and humid atmosphere creates the ideal environment for the growth of microorganisms and moulds during leather items are stored, transported, and used. Therefore, both enterprises and customers are concerned about the antibacterial properties of leather footwear [14]. It is really exciting to have antimicrobial qualities in leather products to overcome this challenge. Numerous compounds and the methods used to provide their antimicrobial properties to leather are not environmentally friendly, harmful to people, and cause bacteria to become resistant to the chemicals [7]. The effectiveness of a wide range of antibacterial and antifungal treatments, including chitosan and its derivatives, polymer compounds containing quaternary ammonium, silver nanoparticles, and zinc oxide nanoparticles, has been investigated in leather treatment [7,[15], [16], [17], [18]]. These substances work by breaking down the cell membranes of bacteria and engaging with them through contact mechanisms [[19], [20], [21]]. Industrial applications have also been explored with synthetic antimicrobial agents such as di-ammonium rings, polyvinyl pyridines, silyl quaternary compounds, chlorinated phenols, quaternary ammonium salts, and polysulfone compounds. Nonetheless, some might be hazardous and poisonous to human health and the environment [22]. Hence, there exists a pressing need for the development of eco-friendly antimicrobial materials that are effective against a broad spectrum of bacteria and moulds [19,23,24].

In recent years, nanotechnology has emerged as a frontier in antimicrobial research, offering innovative solutions to longstanding challenges. The new developments in nanoscale materials have attracted scientists to make strides toward developing materials with better antibacterial features [25]. The antibacterial activity, thermal and electrical insulation, non-toxicity, superior biocompatibility, huge surface area-to-volume ratio, UV blocking capabilities, and photocatalytic activity of magnesium oxide nanoparticles are only a few of their many fascinating characteristics [26]. Magnesium oxide nanoparticle (MgO NP) is a promising alternative to organic and heavy metal-based antimicrobial agents owing to the high rate of digestion in the body. They are not harmful to human health and are effective bactericides as superoxide anion is produced on their surface during their production [27,28]. Inorganic antimicrobial agents (such as MgO) have a high inhibition rate against numerous gram-positive and gram-negative microorganisms, compared to organic antibacterial agents [29]. MgO NPs are highly ionic and have extraordinarily large surface areas and unique crystal morphologies [30]. As a result of the distinctive structures of the nanoscale, MgO possesses novel qualities in the areas of optics, electronics, magnetism, temperature, mechanics, and chemistry [31]. MgO NPs can be produced using ultrasonic synthesis, vapor-liquid-solid synthesis, micro-oven synthesis, colloidal-micelle synthesis, and electrochemical process synthesis [32]. Recently, scientists have been on the hunt for biological alternatives to the outdated physical and chemical synthesis techniques of NP. Producing NPs through biological means is preferred since it is free from harmful substances, cost-effective, non-toxic, and harmless to the environment [33]. Plants are excellent bio-catalysts for the production of NPs and they can actively reduce metal ions and generate functional biocompatible biomolecules [34]. Recent studies have shown that *Azadirachta indica*, *Clitoria ternatea*, *Parthenium* and *Brassica oleracea* can be used as biologically synthesized MgO NPs, which are effective against foodborne pathogens [35].

Chitosan (CS) is a biopolymer produced from chitin, the second most prevalent biopolymer after cellulose. Chitin is obtained through the processes of deproteination and decalcification from crustaceans (such as insects, crabs, and shrimp shells) through extraction using enzymes from fungi [36]. β-(1,4)-2-acetamido-2-deoxy-d'glucopyranose units form a linear polymer in this polysaccharide. Chitosan has the antibacterial feature of being polycationic, which may inhibit bacterial metabolism. Despite the amazing capabilities of chitosan membranes and films, their poor mechanical, thermal, and barrier qualities limit their use in many potential applications like wound dressing and food packaging [26]. Chitosan can help heal wounds because of its similar structure to glycosaminoglycans in the skin [37]. The novel polymers used in food packaging are made from biodegradable ingredients like chitin and lignin [38]. Chitosan, complements the antimicrobial properties of MgO NPs [36,39]. With its cationic nature and biodegradability, chitosan exhibits effective antibacterial activity, making it an ideal candidate for composite materials [36,39]. By harnessing the synergistic effects of MgO NPs and chitosan, it is possible to develop nanocomposites with enhanced antimicrobial properties for applications in leather finishing.

In this research, MgO nanoparticles were prepared using *Aloe vera* leaves, and chitosan was obtained from prawn shells to produce MgO/Chitosan nanocomposite, systematically optimized and characterized the synthesis and application of MgO nanoparticles. The performance of the developed composites as antibacterial agents was investigated using *E. coli* and *B. subtilis* bacteria. The main aim of the research is to develop a green-synthesized MgO/Chitosan nanocomposite and apply it on shoe-lining leather to improve its antibacterial properties against a wide range of microbes evading ecological and environmental problems.

## 2 Experimental procedure

### 2.1 Materials and chemicals

#### 2.1.1 Procurement of materials and chemicals

Fresh *Aloe vera* leaves and prawn shells were procured from the local market in Gulistan, Dhaka, Bangladesh. Chemicals including magnesium nitrate, sodium hydroxide, hydrochloric acid, polyvinyl alcohol, and glacial acetic acid were acquired from local suppliers, specifically From Merk, Germany, for the synthesis of the MgO/chitosan nanocomposite.

### 2.2 Synthesis of MgO/CS nanocomposite

#### 2.2.1 Aloe vera extraction

**Fig. 1:**
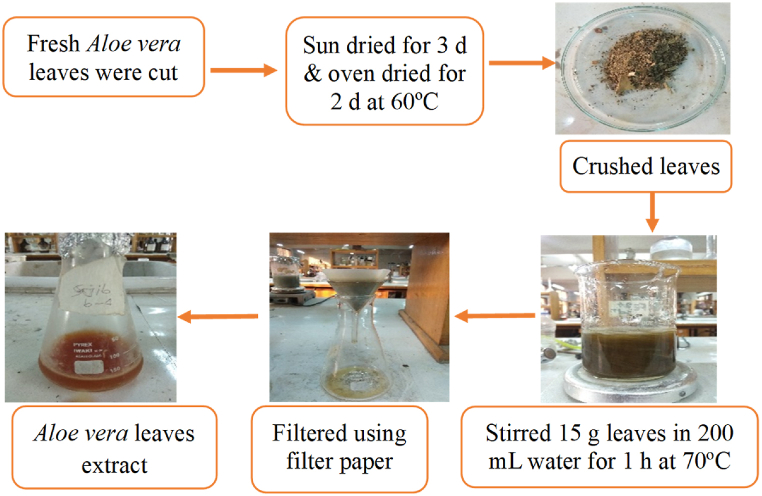
Preparation of *Aloe vera* leaf extract.

This study presents a modified method for *Aloe vera* leaf extract preparation, with slight adjustments to the drying time and temperature compared to the method described by N. Čutović et al. (2023) [40]. Fresh Aloe vera leaves were meticulously collected, washed, and segmented into small portions. Subsequently, they were subjected to a drying process, initially through exposure to sunlight for a period of three days, followed by oven drying at 60 °C for 2 d. The dried and pulverized leaves, weighing 15 g, were mixed with 200 mL of distilled water and subjected to stirring using a magnetic stirrer at 70 °C for 60 min. The resulting solution underwent filtration through Whatman 1 filter paper post-cooling, with the filtrate being refrigerated at 4 °C for subsequent use. The schematic depiction of the preparation of Aloe vera leaf extract is provided in Fig. 1.

#### 2.2.2 Synthesis of MgO nanoparticles

**Fig. 2:**
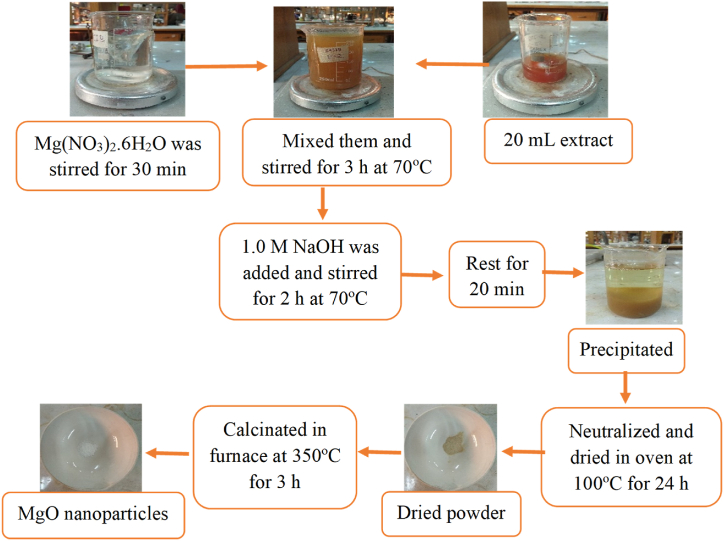
Synthesis of MgO nanoparticles.

As depicted in Fig. 2, the synthesis of MgO nanoparticles entailed a multi-step process involving both natural and synthetic precursors. A 0.01 M magnesium nitrate solution was prepared as a starting point. Subsequently, *aloe vera* extract was gradually introduced into this solution while it was vigorously stirred at 70 °C for a duration of 3 h. This controlled heating and agitation promoted the formation of nanoparticles. To establish an optimal alkaline environment, a 1.0 M NaOH solution was carefully added until the pH reached 10. The stirring process continued at 70 °C for an additional 2 h to ensure complete reaction and uniform nanoparticle distribution, resulting in the precipitation of MgO nanoparticles. Following a 20-min resting period, the precipitate underwent repeated washing with distilled water to eliminate impurities, thus enhancing its purity. To remove residual moisture, the washed precipitate was dried at 100 °C for 24 h before undergoing calcination. The final step involved calcining the dried precipitate at 350 °C for 3 h to refine the nanoparticles, improving their crystallinity and purity. The successful synthesis of MgO nanoparticles was evident from the formation of a white powder, which was carefully preserved for subsequent applications. The chemical reactions during the formation of MgO were as follows.

#### 2.2.3 Extraction of chitosan (CS) from prawn shells

**Fig. 3:**
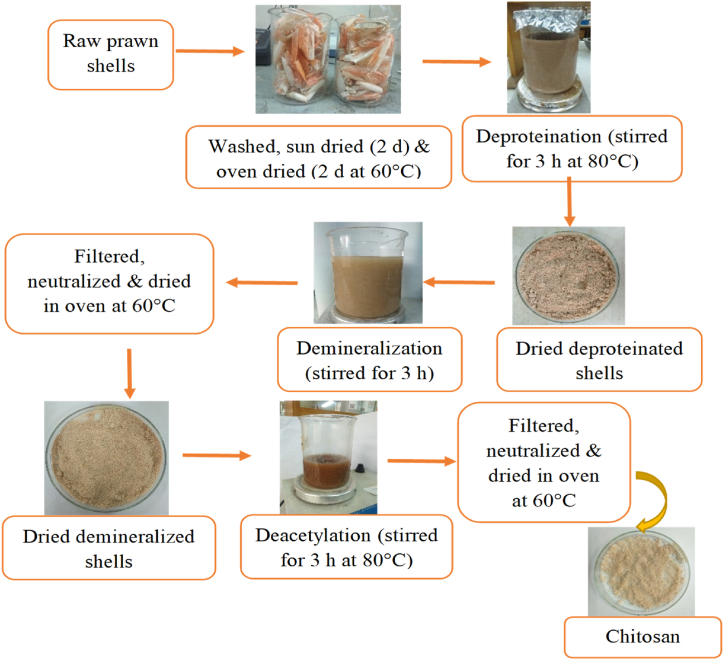
Extraction process of chitosan from prawn shells.

This method is a slight modification of the chitosan extraction process from prawn shells described by Boudouaia et al. [41]. The extraction process of chitosan from prawn shells, as depicted in Fig. 3, began with the thorough cleansing of fresh prawn shells, followed by a sequential drying regimen involving exposure to sunlight for 48 h, followed by additional drying in an air oven at 60 °C for an equivalent duration. Subsequently, the desiccated shells were pulverized, yielding a powdered form that was quantitatively assessed. The extraction of chitosan ensued through a precisely executed three-step procedure, encompassing deproteination, demineralization, and deacetylation.

Deproteination commenced by dissolving 45 g of sodium hydroxide (NaOH) in a 1000 mL beaker containing 900 mL of distilled water, whereupon 50 g of prawn shell powder was incorporated into the solution. This admixture underwent stirring for 3 h at 80 °C, followed by the drying of the shells at 60 °C, with subsequent weight determination to facilitate the subsequent demineralization phase. Demineralization involved the agitation of 522 mL of 3.0 M hydrochloric acid (HCl) with 29 g of deproteinated shells in a 1000 mL beaker for a duration of 3 h. The demineralized shells were then subjected to filtration, followed by a neutralizing wash, drying at 60 °C, and subsequent weight assessment. Deacetylation was initiated by combining 144 g of NaOH with an appropriate volume of distilled water in a 500 mL beaker, followed by continuous agitation to achieve a 288 mL solution. Subsequently, 16 g of demineralized shells were introduced into the beaker, and the mixture was stirred for 3 h at 80 °C. The resultant chitosan product underwent filtration, neutralizing washes, and drying at 60 °C in an oven. The confirmation of chitosan's successful extraction was affirmed through a solubility assay, wherein a small quantity of chitosan was dissolved in a 1.0 % acetic acid solution, demonstrating complete solubility.

#### 2.2.4 Preparation of MgO/CS nanocomposite

**Fig. 4:**
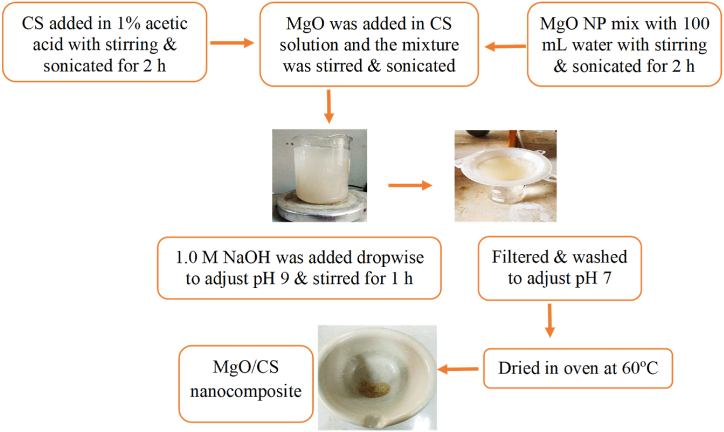
Schematic diagram for preparation of MgO/CS nanocomposite.

Fig. 4 illustrates the preparation of MgO/CS nanocomposite. At first, 1 % acetic acid solution was used to dissolve a certain amount of chitosan, which was then stirred and sonicated for 2 h. Then a specific amount of MgO nanoparticles (following Table 1) were mixed with distilled water (100 mL) and then stirred and sonicated for 3 h. Then the MgO mixture was added drop by drop slowly to the CS solution and then stirred and sonicated for 3 h. Then 1.0 M NaOH was added dropwise with stirring to adjust the pH at 9.0 and continued stirring for 1 h. The mixture was filtered and washed with distilled water to neutralize at pH 7.0. The composite was prepared after 24 h of drying in an air oven at 60 °C. Following the procedure composites (C1, C2, and C3) were prepared in three different ratios of MgO and chitosan (Table 1), optimized the procedure by assessing bacterial growth on lining leather after application of each composite and then characterized.

**Table 1:** Ratio of MgO and chitosan in 3 composites.

Composite ID	MgO (g)	Chitosan (CS) (g)
Composite 1 (C1)	1.0	1.0
Composite 2 (C2)	0.5	1.0
Composite 3 (C3)	1.0	0.5

### 2.3 Characterization of MgO/CS nanocomposite

#### 2.3.1 Fourier transform infrared (FT-IR) spectroscopy

Using an FT-IR spectrometer, (Alpha, USA) with a wavenumber of 4000-400 cm^−1^, MgO nanoparticles, chitosan and MgO/CS nanocomposite were analyzed. The powder samples and the appropriate quantity of KBr were mixed to conduct the test.

#### 2.3.2 Dynamic light scattering (DLS)

DLS is a technique for determining the average size of particles. Brownian motion of nanoparticles causes the scattering of laser beams with varying intensities in a colloidal suspension. Malvern Zetasizer Nano ZS was utilized for the distribution of particle sizes in this study. The analysis utilized deionized water as a dispersion and laser light scattering using an angle of 173°. A temporary folded capillary tube with 1.0 mL of sample was positioned in the instrument's sample holder.

#### 2.3.3 X-ray diffraction (XRD)

XRD is a widely used technique for revealing information about the crystal structure of materials. The crystalline structure of MgO nanoparticles, chitosan, and MgO/CS composite was investigated using a D8 Advance X-Ray Diffractometer made by *Bruker AXS*, Germany. The amplification of XRD patterns 2θ ranges from 5° to 80°.

#### 2.3.4 Thermogravimetric analysis (TGA)

TGA determines the mass change in a sample due to decomposition, desorption, evaporation or sublimation. Thermogravimetric analysis was carried out using thermogravimetric analyzer (SHIMADZU Corp, Japan). Solid state samples were provided in aluminum cells and the cells were subsequently introduced into the machine. The temperature range was 48–700 °C and the heating rate was 10 °C/min.

#### 2.3.5 Scanning electron microscopy (SEM)

Using a scanning electron microscope model-S3000N made by Hitachi, Japan with an acceleration voltage of 15 kV, working distance of 11 mm and spot size of 30, the morphology of MgO nanoparticles was analyzed. The dried samples of MgO nanoparticles were fixed to adhesive carbon tapes supported by metallic disks and their images were captured using the secondary electron (SE) mode at various magnifications.

### 2.4 Application of MgO/CS nanocomposite on the leather surface

The MgO/CS nanocomposite has been applied onto the leather surface using a PVA solution as a binder to form a thin, transparent layer. A 0.25 g of each of the three composites (C1, C2, and C3) was dissolved separately in 25 mL of 0.5 % acetic acid solution. It was then mixed with a 25 mL solution containing 0.25 g of polyvinyl alcohol (PVA). The mixture was stirred for 2 h and each mixed solution was sprayed separately 10 mL per sq. ft. on the semi-chrome lining leather for the assessment of antibacterial effect.

### 2.5 Antibacterial analysis

The lining leather samples coated with composites were tested for antibacterial properties using the agar well diffusion method [42]. *E. coli* (ATCC 11775), and *B. subtilis* (ATCC 6633) bacteria were used for the test. It was proven that these species were present in freshly collected goatskin [43].

### 2.6 Cytotoxicity analysis

The Vero cell line, derived from kidney epithelial cells of an African green monkey, was cultivated in DMEM (Dulbecco's Modified Eagle's Medium), supplemented with 10 % fetal bovine serum (FBS), 1 % penicillin-streptomycin, and 0.25 % gentamycin at the Cell culture laboratory of Centre for Advanced Research in Sciences, University of Dhaka. On a 48-well plate, cells were seeded and cultured at 37 °C with 5 % CO_2_. The next day, a 50 μL autoclaved sample was added. The cytotoxic effects were evaluated 48 h later using an inverted microscope. Each sample was analyzed in a pair of wells.

### 2.7 Physicochemical tests of finished leather

#### 2.7.1 Color rub fastness

SATRA TM 08 method was followed at dry and wet conditions to evaluate the color rub fastness of the leather sample [44]. This likely involves a standardized test simulating real-world friction on leather. The test was performed under both dry and wet conditions, mimicking scenarios like contact with clothing or exposure to moisture. Color rub fastness refers to the leather dye's ability to stay put during such rubbing. To assess this, the rubbed leather and any stains left on a felt material with a standardized gray scale were visually compared. This gray scale assigns a numerical value based on the severity of the color change, allowing for a quantitative evaluation of colorfastness. The color change of the sample and staining on the felt was visually assessed using a gray scale.

#### 2.7.2 Perspiration fastness

To evaluate the perspiration fastness of the leather sample, the IULTCS/IUF 426:2012 (E) method was followed [45]. A perspirometer applies pressure to a leather sample and multifiber fabric, both soaked in alkaline solution. After controlled heating and drying, the multifiber fabric is assessed for color transfer using gray scales. This evaluates how well leather color resists sweat under controlled temperature and humidity.

#### 2.7.3 Water vapor permeability

To determine the water vapor permeability (WVP) of the lining leather, the SATRA TM 172 method was followed. WVP (mg/cm^2^-h) was calculated by using equation (1)

(1) $$\text{WVP}=\frac{\left(M_{2}-M_{1}\right)}{A\;\left(T_{2}-T_{1}\right)}$$

Here, M_2_ = Final mass of the test pot (mg), M_1_ = Initial mass of the test pot (mg), A = Area of the sample (cm^2^) and T_2_ -T_1_ = Time difference (h).

## 3 Results and discussion

### 3.1 Optimization of Aloe vera extract volume

To optimize the *Aloe vera* extract amount, three different quantities (15 mL, 20 mL and 25 mL) of *Aloe vera* extract were used to react with 180 mL of 0.01 M magnesium nitrate solution.

It was observed (Table 2) that the amount of nanoparticle production increased with the addition of *Aloe vera* extract. After the addition of 5 mL of *Aloe vera*, 15.0 mL MgO nanoparticles were increased to 17.5 mg, whereas it was increased to only 1.0 mg for the addition of the same amount of Aloe vera with 20 mL MgO. Therefore, 20 mL *Aloe vera* extract was considered optimum for the production of 74.0 mg MgO nanoparticles.

**Table 2:** Optimization of the amount of *aloe vera* extract.

Volume of precursor solution	Volume of *Aloe vera* extract (mL)	Weight of MgO nanoparticles (mg)
180 mL	15.0	56.5
	20.0	74.0
	25.0	75.0

### 3.2 Characterization

#### 3.2.1 Fourier transform infrared (FT-IR) spectroscopy

**Fig. 5:**
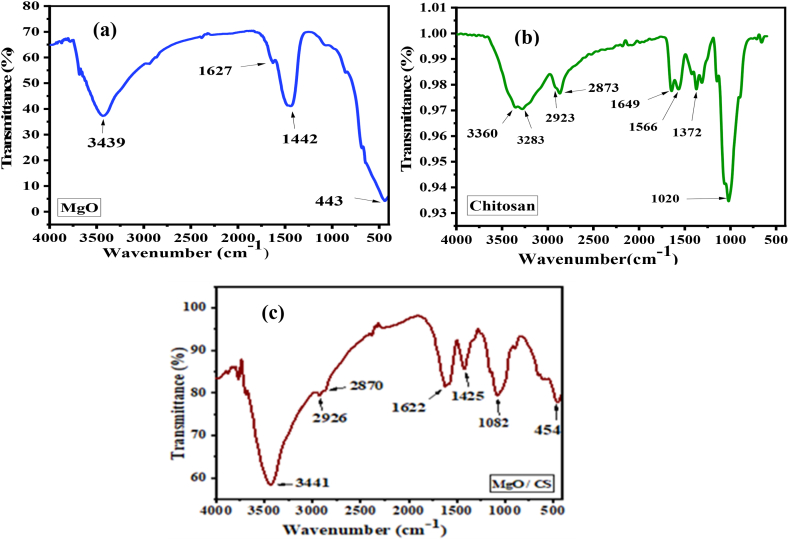
FTIR spectra of extracted (a) MgO nanoparticles, (b) Chitosan and (c) MgO/CS nanocomposite.

In the FTIR spectra of MgO nanoparticles (Fig. 5(a)), water molecules were responsible for the O-H stretching bonds shown at 3439 cm^−1^ and 1627 cm^−1^. The –COO^-^ stretching vibrations, which was chemisorbed onto the MgO surface was attributed to the peak at 1442 cm^−1^. The Mg-O-Mg bond was represented by the peak at 443 cm^−1^ [46]. The FT-IR spectra of the chitosan (Fig. 5(b)) corresponded to O-H and N-H stretching at 3360 cm^−1^ and 3283 cm^−1^. The C-H symmetric and asymmetric stretching peaks was at 2923 cm^−1^ and 2873 cm^−1^. A sharp distinctive stretch of C=O (amide I) and N-H (amide II) was found at 1649 cm^−1^ and 566 cm^−1^ respectively. The symmetrical deformation vibrations of the C-H bond peak was found at 1372 cm^−1^ and C-O stretching sharp peak was observed at 1020 cm^−1^ [47].

Fig. 5. (c) represents the FTIR spectra of the MgO/CS nanocomposite. The peak at 3441 cm^−1^ represented the combined O-H, N-H band and attachment of MgO to the amide groups of chitosan. The absorption peaks at 2926 cm^−1^ and 2870 cm^−1^ were attributable to the stretching vibration of C-H in the chitosan polymer. The C-N axial deformation (amine group band) was found at 1425 cm^−1^ peak. The stretching vibration of the C-O group was detected at 1622 cm^−1^ and 1082 cm^−1^. The peak at 454 cm^−1^ was recognized as vibration of Mg-O-Mg bonds [48]. The characteristic peaks of FTIR had confirmed the successful integration of MgO nanoparticles into the CS matrix.

#### 3.2.2 Dynamic light scattering

**Fig. 6:**
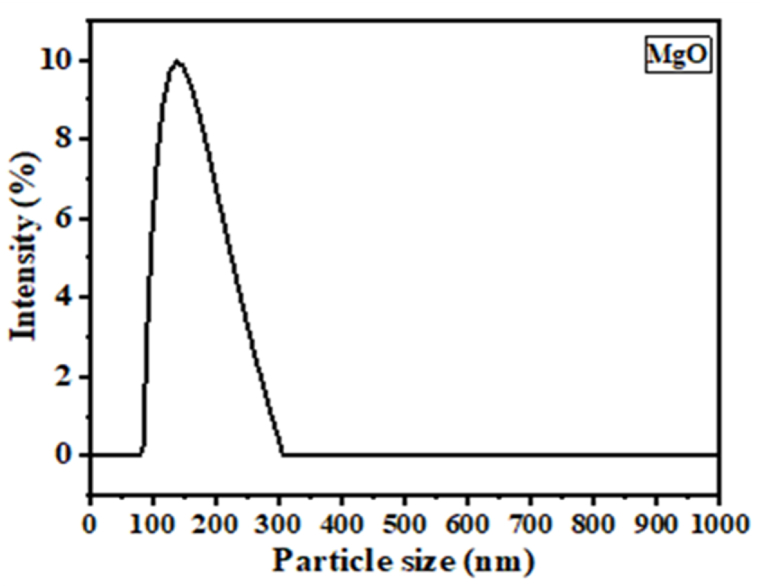
Dynamic light scattering analysis of MgO nanoparticles.

The average particle size of the produced MgO nanoparticles was determined using the dynamic light scattering technique (Fig. 6). The measured particle size was 136 nm with a polydispersity index of 25.2 %.

#### 3.2.3 X-ray diffraction (XRD)

**Fig. 7:**
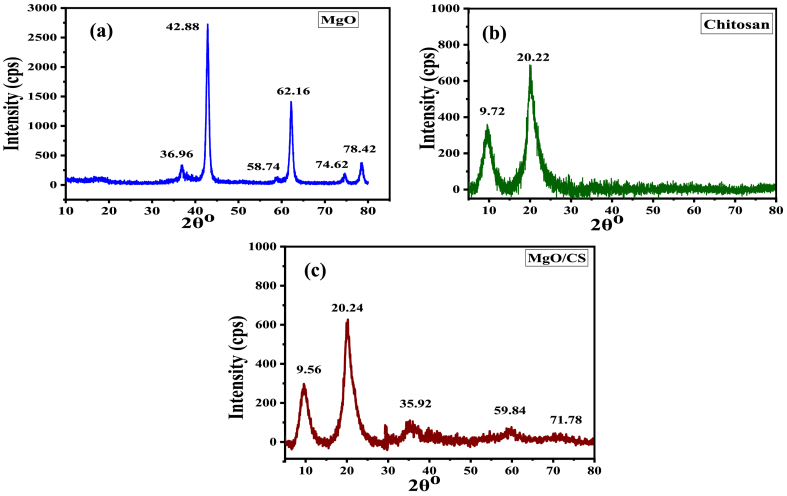
X-ray diffraction analysis of (a) MgO nanoparticles, (b) Chitosan and (c) MgO/CS.

X-ray diffraction analysis of MgO nanoparticles, Chitosan and MgO/CS is shown in Fig. 7. Pure MgO nanoparticles exhibited characteristic peaks at 36.96^o^, 42.88^o^, 58.74^o^, 62.16^o^, 74.62^o^ and 78.42^o^ on the XRD pattern. It was represented that the nanoparticles were extremely crystalline structures [49]. The Debye-Scherrer equation (D = Kλ/βcosθ) was used to calculate the crystallite size diameter of MgO nanoparticles, where D is the crystallite size diameter, β is the FWHM (full-width at half-maximum or half-width) in radians, θ is the position of the maximum diffraction peak, K is the so-called shape factor, which typically has a value of about 0.94. The crystallite size of MgO nanoparticles was found 10.3 nm [50].

The broad diffraction peaks at 9.72 and 20.22^o^ were seen in the XRD pattern of prawn shells extracted from chitosan; these peaks indicated the semi-crystalline structure of chitosan [51]. The XRD pattern of MgO/CS exhibits broad peaks at 2θ = 9.56 and 20.24^o^ representing the semi-crystalline chitosan. In addition to the broad peaks, it had diffraction peaks at 35.92, 59.84, and 71.78^o^, which corresponded to the cubic lattice of MgO. Contrary to the MgO nanoparticles, the peak at 42.88 and 62.16^o^ was not observed in the MgO/CS XRD pattern. The crystallinity of MgO/CS was lower than that of MgO nanoparticles. The results suggested that the MgO nanoparticle was successfully diffused throughout the CS matrix to form the MgO/CS nanocomposite [46].

#### 3.2.4 Thermogravimetric analysis

**Fig. 8:**
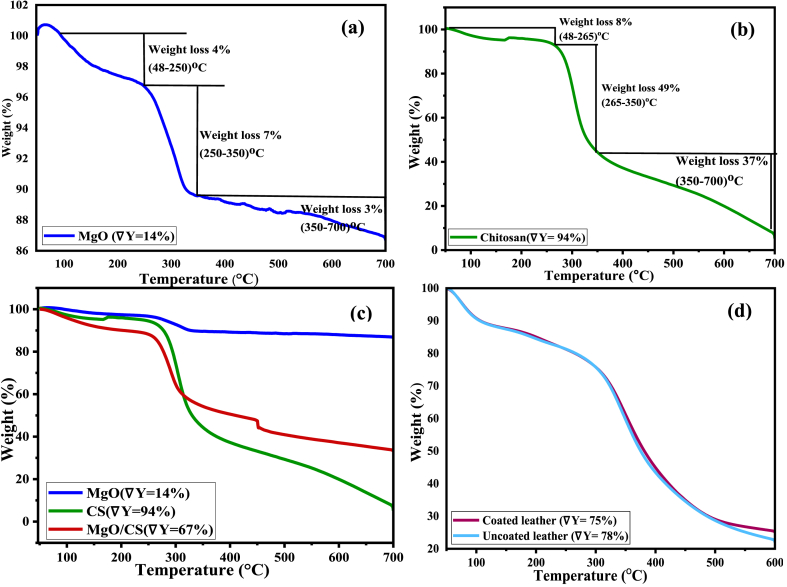
TGA curve of (a) MgO nanoparticle, (b) Chitosan, (c) MgO, CS and MgO/CS and (d) uncoated and coated leather.

According to the TGA curve (Fig. 8(a)), weight loss of MgO nanoparticle begins around 100 °C due to the evaporation of water. The majority of weight loss occurred between 250 and 350 °C, indicating a loss of approximately 7 % of weight due to the disintegration and removal of organic groups present in the sample. There was a 3 % weight loss from 350 to 700 °C. Therefore, it results in a total 14 % weight loss from 48 to 700 °C [52]. Fig. 8(b) depicts the thermograms of chitosan at a scan rate of 10 °C per minute. The sample loses around 6 % of its weight from 48 to 250 °C owing to evaporation of water absorbed by the chitosan sample.

Due to dehydration, deacetylation and depolymerization, the chitosan backbone degrades thermally between 250 and 400 °C, resulting in the greatest weight loss [53]. Fig. 8(c) shows that the incorporation of MgO nanoparticles in chitosan increased its thermal stability. The weight loss of MgO/CS nanocomposite is 67 %, which was 94 % for chitosan before the incorporation of MgO. The uncoated leather shows a net weight loss of about 78 % from 48 to 700 °C. However, the coated leather shows 75 % weight loss in the same temperature range. Hence, the thermal stability is increased with the coating of MgO/CS nanocomposite.

#### 3.2.5 Scanning electron microscopic (SEM) analysis

**Fig. 9:**
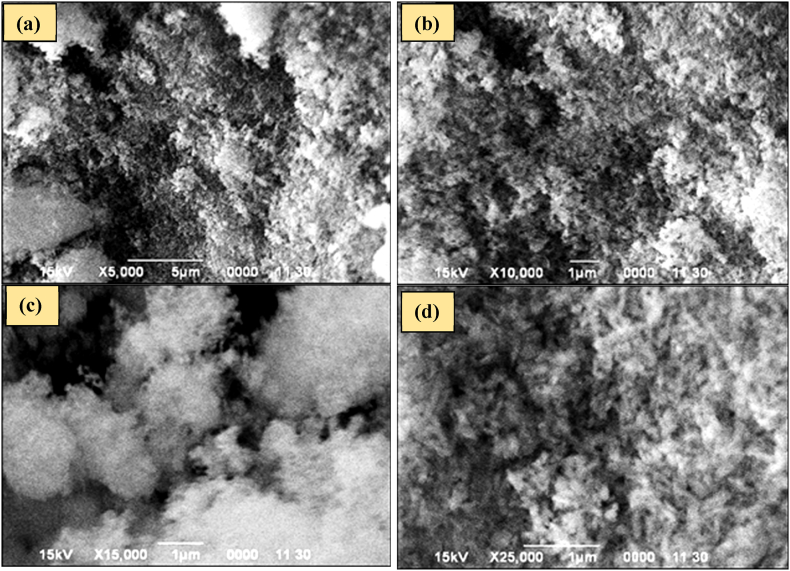
SEM images of MgO nanoparticles (a) × 5000, (b) × 10000, (c) × 15000 and (d) × 25000.

The morphologies of produced MgO nanoparticles were investigated by SEM at various magnifications (Fig. 9). It was exhibited a non-uniform distribution of spherical particles and was found as either a single particle or a cluster of particles. It is indicated that the particles are moderately aggregated. The surface of MgO nanoparticles was studied and it was shown that the aggregated developments have a significant amount of surface roughness [54].

### 3.3 Antibacterial analysis of leather

**Fig. 10:**
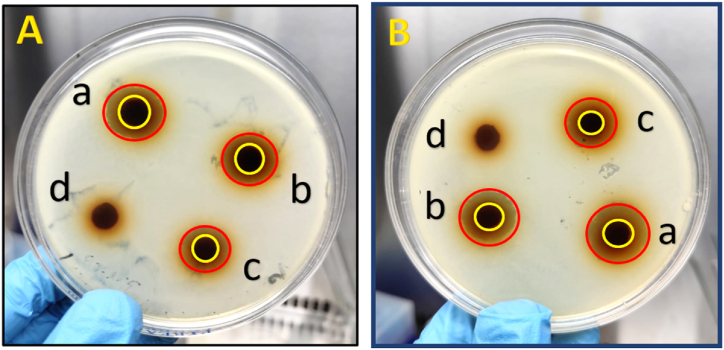
Antibacterial test on Agar plate depicted by non-growth zones (marked in red circle) and leather sample (marked in yellowish circle) using (A) *B. subtilis* and (B) *E. coli* on leather (a) C1, (b) C2, (c) C3 and (d) unfinished leather.

The release of Mg^2+^ ions and physical contact with bacterial cells are two aspects of the MgO/CS composite's antibacterial process. Furthermore, MgO nanoparticles produce reactive oxygen species (ROS), which cause bacterial cells to experience oxidative stress. Through nutritional restriction and electrostatic interactions, chitosan enhances antibacterial activity. By focusing on various cellular components and reducing the possibility of bacterial resistance, the synergistic actions of MgO nanoparticles and chitosan improve overall antibacterial effectiveness [55]. Antimicrobial testing involves evaluating the effectiveness of antimicrobial agents by exposing representative strains of gram-negative (*E. coli*) and gram-positive (*B. subtilis*) bacteria. The effectiveness of the antibacterial properties of MgO/CS nanocomposites was evaluated using the zone of inhibition technique (Fig. 10) by applying to the lining leather. *E. coli* (ATCC 11775), and *B. subtilis* (ATCC 6633) were used for the test. Composites 1, 2 and 3 were applied individually to 3 pieces of leather. The unfinished leather showed no inhibition zone. The nanorods have a diameter of 46 nm and a length of 185 nm. With these dimensions, MgO displays good antibacterial activity with a zone of inhibition of 19 mm against *V. cholerae* and 29 mm against *S. flexneri* [56].

Table 3 presents the mean inhibition zone and indicated that composite 1 had the highest inhibition zone against *E. coli* and *B. subtilis*. It was revealed that composite 1 provides a higher antibacterial property to the leather than composites 2 and 3 (Fig. 10 and Table 3). Therefore, only composite 1 was characterized and used for investigation and study.

**Table 3:** Inhibition zones of leather samples.

Sample	Inhibition zone (mm)		
	*E. coli*	*B. subtilis*	Control (Chloramphenicol-30 μg)
Composite 1	13	12	28
Composite 2	12	10	28
Composite 3	10	9	28

### 3.4 Cytotoxicity analysis

Cytotoxicity testing is a crucial evaluation conducted to assess the potential adverse effects of materials on living cells. This test is particularly important in biomedical and biotechnological applications to ensure the safety of materials intended for use in contact with biological systems. The cytotoxic effects were evaluated 48 h later using an inverted microscope. Simultaneously, a test was done with distilled water which was considered as control. It was observed that greater than 95 % of Vero cells survived on the plate of MgO/CS, which was similar to the control.

The control group, consisting of distilled water, exhibited a survival rate of greater than 95 %. This observation indicates that no cytotoxicity was observed on the Vero cell line when exposed to distilled water, reaffirming its suitability as a neutral control. Similarly, both the MgO/Chitosan nanocomposite and the MgO/Chitosan sample after wash exhibited survival rates exceeding 95 %. This was suggested that neither the nanocomposite nor the washing process induced significant cytotoxic effects on the Vero cell line. The high survival percentage implies that the nanocomposite material is biocompatible and does not adversely affect cell viability under the experimental conditions.

**Fig. 11:**
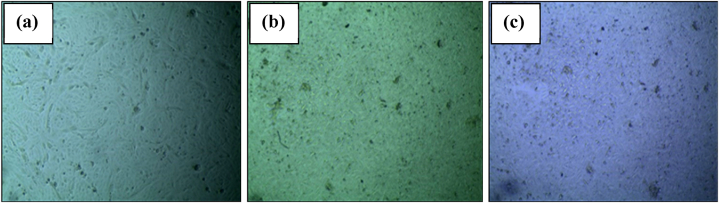
Cytotoxicity analysis of MgO/CS (a) Control, (b) MgO/CS, and (c) MgO/CS after wash.

Fig. 11 and Table 4 ensured that the C1 composite can be considered as non-cytotoxic.

**Table 4:** Survival percentage of Vero cells.

Sample	Survival of Vero cells	Remarks
Control (distilled water)	> 95 %	No cytotoxicity was observed on Vero cell line.
MgO/Chitosan	> 95 %	
MgO/Chitosan after wash	> 95 %	

### 3.5 Physicochemical properties of finished leather

#### 3.5.1 Color rub fastness

The uncoated, PVA-coated and MgO-CS coated leather performed the same in dry color rub fastness (Table 5). Therefore, the composite had no negative effect on the color properties of the leather. The acceptable standard value of the gray scale rating is 3–5.

**Table 5:** Dry and wet color rub fastness of uncoated and MgO/CS coated lining leather.

Leather type	Sample	Gray scale rating (Dry)		Gray scale rating (Wet)	
		512 cycles	1024 cycles	512 cycles	1024 cycles
Uncoated	Leather	5	5	4	3/4
	Felt	5	5	3	2/3
PVA coated	Leather	5	5	3/4	3
	Felt	5	5	2/3	2
PVA + MgO-CS coated	Leather	5	5	4/5	4
	Felt	5	5	4	3

In case of wet color rub fastness, the uncoated leather showed less fastness property than the MgO/CS coated leather (Table 5). The MgO/CS coating protected the leather surface reducing the tendency of color migration to the cotton felt and increased the wet color rub fastness.

#### 3.5.2 Perspiration fastness

The MgO/CS coating on the leather surface slightly increased the perspiration fastness of the lining leather. The grain surfaces of the MgO/CS-coated and uncoated leather showed similar results (Table 6). Perspiration resistance is slightly reduced by the water-soluble PVA coating. However, different fibers in the standard multifiber cloth had better fastness properties as the layer of PVA and composite lessened the contact of leather with perspiration.

**Table 6:** Perspiration fastness of uncoated and MgO/CS coated lining leather.

Leather type	Sample	Gray scale rating						
		Standard Multifiber cloth						Leather
		Cellulose	Cotton	Nylon	Polyester	Acrylic	Wool	
Uncoated	Grain	4	4	4	3/4	4	4	5
	Flesh	2/3	3/4	3/4	3	3	2/3	5
PVA coated	Grain	4	3/4	3/4	4	3/4	3/4	4/5
	Flesh	2/3	2/3	2/3	3	2	2/3	4/5
PVA + MgO/CS coated	Grain	4	3/4	4	4	4/5	3/4	5
	Flesh	2/3	3/4	3/4	3	3	2/3	4/5

#### 3.5.3 Water vapor permeability (WVP)

**Fig. 12:**
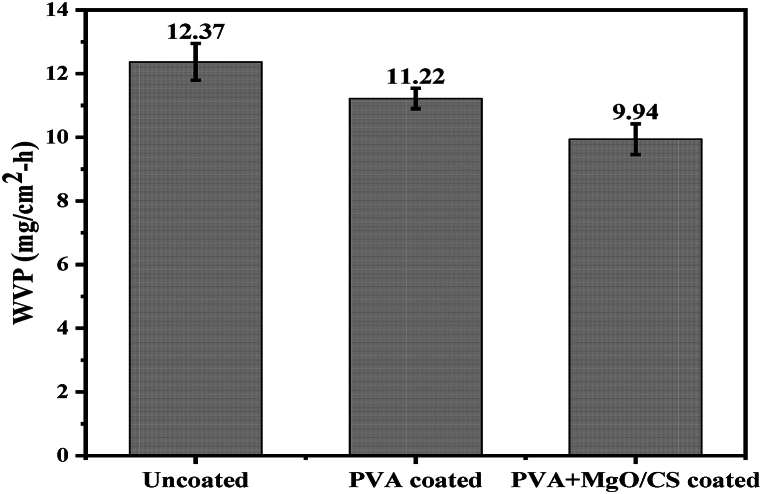
Water vapor permeability of uncoated and coated leather.

The water vapor permeability (WVP) of uncoated, PVA-coated and PVA + MgO/CS coated leather was 12.37, 11.22 and 9.94 mg cm^−2^.hr^−1^ (Fig. 12). It is observed that the coating of PVA and PVA + MgO/CS composite reduced the water vapor permeability from 12.37 to 9.94 mg cm^−2^.hr^−1^. However, all the values were much higher than the required standard value (minimum 2 mg cm^−2^.hr^−1^) of lining leather.

## 4 Conclusions

The antibacterial characteristics of lining leather are significantly improved by the addition of MgO/CS nanocomposite, as demonstrated by the considerable inhibition zones of 13 mm and 12 mm against *B. subtilis* and *E. coli*, respectively. The effective integration of the produced materials, such as MgO nanoparticles, chitosan, and MgO/CS, onto the leather surface has been confirmed by a thorough characterization using methods including FTIR, XRD pattern, SEM, and TGA analyses. When the MgO/CS nanocomposite was applied with a PVA solution as a binder, a thin layer was formed that provided good antibacterial activity without compromising the leather's key properties. Furthermore, measured figures revealed that the coated leather's water vapor permeability decreased from 12.37 mg cm^−2^.hr^−1^ to 9.94 mg cm^−2^.hr^−1^, which was still well within acceptable limits for lining leather. Even with that slight decrease, the leather's efficiency was unaffected, making it appropriate for uses. One of the main drawbacks and limitations of the study is that the industry-level bulk production was not practiced. However, MgO/CS has a significant potential to enhance antibacterial qualities, which implies a wide range of uses in the leather production industry. Future research should investigate how to use its antibacterial activity to include it in various leather kinds with a range of finishing techniques. To fully evaluate its antibacterial characteristics, it is also necessary to test its efficacy against other bacteria and fungi. Additional research should be done to address the decreases in water vapor permeability and to compare it with industry-produced lining leather; one such approach is to combine chitosan and finishing materials with different safe metal and metal oxide nanoparticles. This approach offers a viable way to improve antimicrobial efficacy without compromising material functionality or integrity, guaranteeing the leather goods' continual relevance and usability across a range of industries.

## CRediT authorship contribution statement

**Sobur Ahmed:** Writing – review & editing, Writing – original draft, Visualization, Validation, Supervision, Project administration, Methodology, Funding acquisition, Conceptualization. **Sajib Sarker Imon:** Writing – original draft, Visualization, Software, Methodology, Investigation, Formal analysis, Data curation. **Md Jawad Hasan:** Writing – review & editing, Methodology, Investigation, Formal analysis, Data curation. **Md Samaul Alam:** Writing – review & editing, Methodology, Investigation, Formal analysis, Data curation.

## Data availability

Data will be made available on request.

## Declaration of competing interest

The authors declare that they have no known competing financial interests or personal relationships that could have appeared to influence the work reported in this paper.
